# Supplementary material for: Early Colonization of the Intestinal Microbiome of Neonatal Piglets Is Influenced by the Maternal Microbiome
Source: Animals (Basel). 2023 Oct 31;13(21):3378. doi: 10.3390/ani13213378 (PMC10650534; doi:10.3390/ani13213378)
Supplement: Supplementary file 1 [file animals-13-03378-s001.zip › animals-2635487-supplementary.pdf]

Supplementary Table S1. Raw reads, High quality sequences and OTUs from six groups.

Group	Raw reads	High quality sequences	OTUs
1d	4,784,260	4,145,311	9,685
7d	4,643,977	3,809,764	2,392
14d	4,062,590	3,303,840	2,998
21d	3,708,105	3,046,006	4,031
28d	3,553,611	2,918,794	5,756
Sow	3,653,236	3,073,906	7,480

Supplementary Table S2. Core bacterial taxa (genus level) were present in the intestines of all piglets during the first 28 d after birth.

	Number of OTU (mean ± SD)				
Taxonomy(genus)	day1	day7	day14	day21	day28
Clostridium_sensu_stricto_1	23026.43 ± 6179.53	2352.62 ± 2369.16	1999.57 ± 1972.76	482.21 ± 424.56	1722.19 ± 2258.07
Turicibacter	8047.95 ± 3127.88	4430.05 ± 5054.85	6776.62 ±5415.54	1030.02 ± 1502.84	1119.62 ± 1543.49
Romboutsia	6618.88 ± 2370.55	9272.86 ± 5706.36	3136.93 ± 4462.64	5081.10 ± 4534.92	1349.02 ± 783.27
Streptococcus	2705.60 ± 2045.51	6409.21 ± 7633.78	2872.21 ± 6238.04	3117.24 ± 2953.47	346.76 ± 449.65
Bacteroides	533.64 ± 421.33	1250.98 ± 1869.97	303.24 ± 272.08	960.71 ± 1200.77	1262.29 ± 1299.15

Supplementary Table S3. Microbiome (at the genus level) is transmitted vertically from sows to piglets.

Number of OTU about vertical transmission microbe sow to piglet					
Phylum	Genus	1-7d	7-14d	14-21d	21-28d
Actinobacteriota	Corynebacterium	15	10	10	2
	Trueperella	5	2	2	2
	Rothia	5	4	2	1
	Actinomyces	3	1	1	1
	Bifidobacterium	2	2	2	1
	Dietzia	2	1	1	1
	Brevibacterium	1	1	1	1
	Atopobium	1	1	1	1
	Olsenella	1	1	1	1
	Collinsella	1	1	1	1
	Enterorhabdus	1	1	1	1
	Brachybacterium	2	1	0	0
	Kocuria	2	2	1	0
	Eggerthella	2	2	1	0
	Arcanobacterium	1	0	0	0
	Gordonia	1	0	0	0
	Blastococcus	1	0	0	0
	Ornithinimicrobium	1	0	0	0
	Glutamicibacter	1	1	1	0
	Nesterenkonia	1	1	0	0
	Cutibacterium	1	1	0	0
	Propioniciclava	1	0	0	0

	Eggerthellaceae__DNF00809	1	1	1	0
Bacteroidota	Muribaculaceae	31	23	20	20
	Bacteroides	32	25	21	17
	Prevotella	27	22	15	14
	Rikenellaceae-RC9-gut_group	22	16	16	12
	Alloprevotella	9	7	6	6
	Parabacteroides	8	6	5	5
	Prevotellaceae_NK3B31_group	10	4	3	3
	Prevotellaceae_UCG-001	3	3	3	3
	Alistipes	3	3	3	3
	Porphyromonas	12	9	2	1
	Bergeyella	9	7	3	1
	p-251-o5	3	2	2	1
	Prevotellaceae_UCG-003	3	1	1	1
	Butyricimonas	1	1	1	1
	Muribaculaceae__CAG-873	1	1	1	1
	p-2534-18B5_gut_group	1	1	1	1
	Prevotellaceae_UCG-004	1	1	1	1
	Rikenellaceae__dgA-11_gut_group	1	1	1	1
	Bacteroidales_RF16_group	6	4	0	0
	Chryseobacterium	5	3	2	0
	Myroides	4	2	0	0
	Sphingobacterium	4	1	0	0
	Empedobacter	3	1	1	0
	F082	1	1	0	0
	Prevotellaceae_YAB2003_group	1	0	0	0

	Rikenellaceae__DMER64	1	0	0	0
	Rikenellaceae__U29-B03	1	1	0	0
	Flavobacterium	1	0	0	0
	Soonwooa	1	1	0	0
Campilobacterota	Campylobacter	6	3	3	3
	Arcobacter	2	2	2	1
	Helicobacter	1	0	0	0
Chloroflex	JG30-KF-CM45	1	1	1	0
Cyanobacteria	Chloroplast	6	4	1	1
Desulfobacterota	Bilophila	1	1	1	1
	Bradymonadales	1	1	1	1
	Desulfovibrio	1	0	0	0
Euryarchaeota	Methanobrevibacter	4	3	3	3
	Methanosphaera	1	1	1	1
Fibrobacterota	Fibrobacter	1	0	0	0
Firmicutes	Christensenellaceae_R-7_group	28	19	17	17
	Lactobacillus	24	23	20	11
	Clostridium_sensu_stricto_1	46	32	21	9
	Oscillospiraceae__UCG-002	12	11	10	8
	Lachnoclostridium	8	8	7	7
	Staphylococcus	25	19	13	6
	Streptococcus	16	14	10	6
	[Eubacterium]-coprostanoligenes-group	14	12	9	6
	Oscillospiraceae__NK4A214_group	9	8	8	6
	Blautia	8	7	6	6
	Oscillospiraceae__UCG-005	17	12	7	5

Phascolarctobacterium	7	6	6	5
Subdoligranulum	6	4	4	4
Anaerovoracaceae__Family-XIII-AD3011-group	6	4	4	4
Ruminococcus	11	6	5	3
Clostridia_UCG-014	7	5	5	3
Enterococcus	6	6	5	3
Faecalibacterium	5	3	3	3
Anaerococcus	5	3	3	3
Coprococcus	4	4	3	3
Butyricicoccus	3	3	3	3
Peptococcus	3	3	3	3
UCG-010	9	3	3	2
RF39	5	3	2	2
Weissella	4	4	2	2
Roseburia	4	2	2	2
Peptostreptococcus	4	3	2	2
Solobacterium	3	2	2	2
Aerococcus	3	2	2	2
[Eubacterium]-hallii-group	3	2	2	2
Butyricicoccaceae__UCG-008	3	3	2	2
Anaerovibrio	3	2	2	2
Veillonella	3	2	2	2
[Clostridium]-innocuum_group	2	2	2	2
Holdemanella	2	2	2	2
Clostridium_sensu_stricto_6	2	2	2	2
Tyzzerella	2	2	2	2

Colidextribacter	2	2	2	2
Fournierella	2	2	2	2
Incertae-Sedis	2	2	2	2
Megasphaera	2	2	2	2
Terrisporobacter	9	4	3	1
Helcococcus	7	5	3	1
Clostridia_vadinBB60_group	6	4	1	1
Peptoniphilus	5	4	4	1
Selenomonas	5	4	2	1
Erysipelatoclostridiaceae__UCG-004	4	1	1	1
Jeotgalicoccus	4	2	1	1
Candidatus_Soleaferrea	4	3	1	1
Sharpea	3	1	1	1
Ignavigranum	3	1	1	1
Dorea	3	2	1	1
Marvinbryantia	3	1	1	1
Mogibacterium	3	1	1	1
Romboutsia	3	2	1	1
Kurthia	2	2	1	1
Catenisphaera	2	1	1	1
Facklamia	2	1	1	1
Jeotgalibaca	2	1	1	1
Mycoplasma	2	2	1	1
[Ruminococcus]-torques-group	2	2	2	1
Acetitomaculum	2	1	1	1
Epulopiscium	2	1	1	1

	Lachnospiraceae_NK4A136_group	2	2	1	1
	[Eubacterium]-brachy-group	2	1	1	1
	Lysinibacillus	1	1	1	1
	Sporosarcina	1	1	1	1
	Catenibacterium	1	1	1	1
	Erysipelatoclostridium	1	1	1	1
	[Anaerorhabdus]-furcosa_group	1	1	1	1
	Faecalicoccus	1	1	1	1
	Turicibacter	1	1	1	1
	Pediococcus	1	1	1	1
	Lactococcus	1	1	1	1
	[Eubacterium]-fissicatena-group	1	1	1	1
	[Ruminococcus]-gauvreauii-group	1	1	1	1
	Agathobacter	1	1	1	1
	Anaerostipes	1	1	1	1
	Eisenbergiella	1	1	1	1
	Howardella	1	1	1	1
	Hungatella	1	1	1	1
	Lachnospiraceae_AC2044_group	1	1	1	1
	Lachnospiraceae_ND3007_group	1	1	1	1
	Lachnospiraceae_XPB1014_group	1	1	1	1
	Flavonifractor	1	1	1	1
	Intestinimonas	1	1	1	1
	Pseudoflavonifractor	1	1	1	1
	Hydrogenoanaerobacterium	1	1	1	1
	[Eubacterium]-siraeum-group	1	1	1	1

Negativibacillus	1	1	1	1
Ruminococcaceae__UBA1819	1	1	1	1
Intestinibacter	1	1	1	1
Finegoldia	1	1	1	1
Mitsuokella	1	1	1	1
Negativicoccus	1	1	1	1
Oscillibacter	5	3	0	0
[Ruminococcus]-gnavus-group	4	4	3	0
Peptostreptococcales-Tissierellales__W5053	4	2	2	0
Gemella	3	3	1	0
Cellulosilyticum	3	2	2	0
Dialister	3	2	0	0
Erysipelotrichaceae_UCG-003	2	0	0	0
Erysipelothrix	2	0	0	0
Globicatella	2	2	2	0
Vagococcus	2	0	0	0
Mageibacillus	2	2	0	0
Clostridium_sensu_stricto_13	2	2	1	0
Clostridium_sensu_stricto_2	2	2	1	0
Moryella	2	1	0	0
Shuttleworthia	2	1	1	0
Anaerovoracaceae__Family-XIII-UCG-001	2	2	0	0
Clostridioides	2	2	2	0
_Proteocatella	2	0	0	0
Gallicola	2	1	0	0
Parvimonas	2	1	0	0

	Savagea	1	0	0	0
	Dielma	1	1	1	0
	Aerosphaera	1	0	0	0
	Alloiococcus	1	1	1	0
	Atopostipes	1	1	1	0
	Macrococcus	1	1	1	0
	Nosocomiicoccus	1	1	1	0
	Proteiniclasticum	1	0	0	0
	Pseudoramibacter	1	0	0	0
	[Eubacterium]-eligens-group	1	0	0	0
	Lachnospiraceae_FCS020_group	1	0	0	0
	Lachnospiraceae_UCG-004	1	0	0	0
	Lachnospiraceae_UCG-007	1	1	1	0
	Oribacterium	1	1	1	0
	Monoglobus	1	0	0	0
	Oscillospira	1	1	1	0
	Anaerotruncus	1	1	1	0
	Pygmaiobacter	1	0	0	0
	Guggenheimella	1	1	1	0
	Acidaminococcus	1	0	0	0
	Schwartzia	1	0	0	0
Fusobacteriota	Fusobacterium	7	5	5	5
	Leptotrichia	2	2	2	0
	Streptobacillus	1	1	1	0
Patescibacteria	Gracilibacteria	1	1	1	0
	Candidatus_Saccharimonas	1	1	0	0

	Saccharimonadaceae__TM7a	1	1	1	0
	Saccharimonadales	1	1	1	0
Planctomycetota	Pirellulaceae__p-1088-a5_gut_group	2	0	0	0
Proteobacteria	Moraxella	14	11	7	5
	Succinivibrio	3	2	2	2
	Escherichia-Shigella	2	2	2	2
	Proteus	2	2	2	2
	Pasteurella	2	2	2	2
	Actinobacillus	19	14	3	1
	Sutterella	2	1	1	1
	Klebsiella	2	2	1	1
	Succinivibrionaceae_UCG-001	1	1	1	1
	Enhydrobacter	1	1	1	1
	Acinetobacter	16	10	7	0
	Neisseria	5	4	3	0
	Psychrobacter	4	4	4	0
	Pseudomonas	3	2	1	0
	Thiopseudomonas	3	1	1	0
	Methylobacterium-Methylorubrum	2	0	0	0
	Mitochondria	2	1	0	0
	Comamonas	2	2	2	0
	Pseudochrobactrum	1	1	1	0
	Bradyrhizobium	1	0	0	0
	Novosphingobium	1	0	0	0
	Alcaligenaceae	1	1	1	0
	Uruburuella	1	1	1	0

	Ignatzschineria	1	0	0	0
	Koukoulia	1	0	0	0
	Marinospirillum	1	1	1	0
	Oblitimonas	1	1	1	0
	Luteimonas	1	1	0	0
Spirochaetota	Treponema	17	12	9	6
	Sphaerochaeta	1	1	0	0
Synergistota	Cloacibacillus	1	0	0	0
	Pyramidobacter	1	0	0	0
Verrucomicrobiota	WCHB1-41	5	2	1	1
	Akkermansia	2	2	1	0
